# Successful Use of the ŌNŌ Retrieval Device for Stent Removal in a High-Risk Pediatric Patient: Case Report

**DOI:** 10.1016/j.jscai.2025.103812

**Published:** 2025-08-19

**Authors:** Avram R.P. Rago, John Wiegand, James A. Thompson

**Affiliations:** a Department of Pediatrics, University of South Florida Morsani College of Medicine, Tampa, Florida; b Division of Pediatric Cardiology, Johns Hopkins All Children’s Hospital, St. Petersburg, Florida

**Keywords:** case reports, congenital heart disease, foreign body retrieval, new devices, pediatric intervention

## Abstract

The management of misplaced or embolized devices within the cardiovascular system is critical in interventional cardiology. While uncommon, embolized material can cause significant morbidity. The ŌNŌ retrieval device has been used for retrieval of endovascular material in adults and pediatric patients. In this report, we describe the youngest patient to date who has undergone endovascular material removal using the ŌNŌ device.

## Introduction

The ŌNŌ retrieval device (ŌNŌCOR) is a novel transcatheter device designed to capture and remove material from the cardiovascular system. In this case report, we describe the youngest known patient to date who has undergone endovascular material removal using the ŌNŌ device. Before our case, the youngest known patient in whom the ŌNŌ device was used was a 14-year-old boy with a subacute intracardiac thrombus.^1^

## Case report

A 4-month-old boy born at 34 weeks’ gestation with L-transposition of the great arteries, pulmonary atresia, atretic right-sided atrioventricular valve, and bilateral superior vena cava underwent stenting of the patent ductus arteriosus in the neonatal period as the primary palliation before the planned Glenn anastomosis.

**Figure 1:**
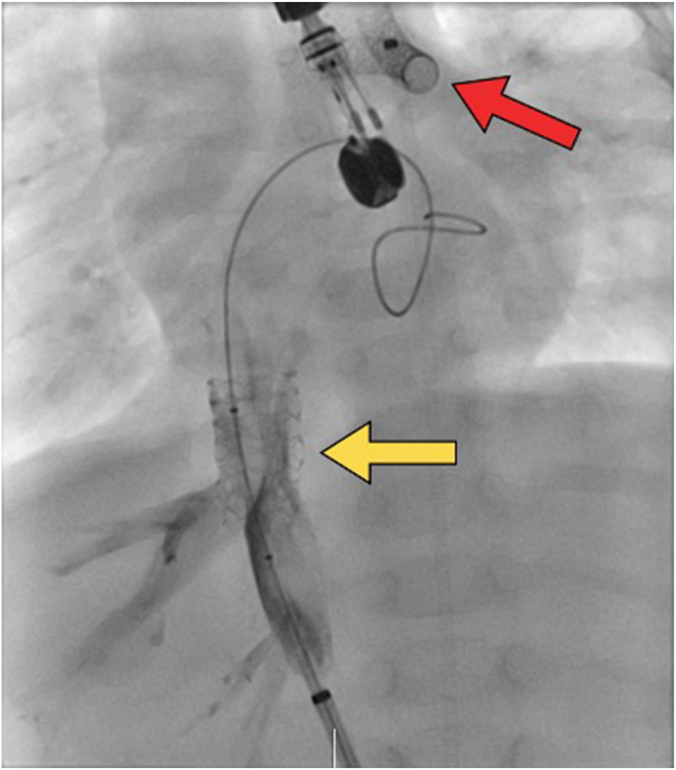
**The dislodged atrial septal defect stent is shown parked in the inferior vena cava (yellow arrow).** The patent ductus arteriosus stents are also visualized (red arrow).

He did well and was discharged home to follow-up with his primary cardiologist. At 3 months of age, he presented to an outside hospital with worsening hypoxemia in the setting of multiple respiratory viruses. He was transferred to our center for further evaluation and management. On hospital day 5, he had an acute decompensation with desaturations and hypotension requiring bag-valve mask ventilation, fluid resuscitation, and epinephrine. He was taken immediately to the catheterization laboratory and was found to have significant neointimal hyperplasia within his ductal stent. Therefore, he underwent further stent placement and dilation with 4.5-mm × 18.0-mm and 4.5-mm × 15.0-mm Onyx Frontier stents (Medtronic). Incidentally, at that time, he was found to have a 4-mm Hg gradient across his atrial septum with an A-wave of 17 mm Hg in the right atrium. An echocardiogram demonstrated a pinhole opening in the atrial septum with significant right to left bowing. An 8.0-mm × 24.0-mm Genesis premounted stent (Cordis) was placed across the atrial septum. Almost immediately, the stent dislodged and migrated into the right atrium. The stent was unable to be pushed back across the atrial septum. Given the difficulty of retrieval at this time, the decision was made to position the stent in the inferior vena cava (IVC) with a plan of having the stent removed surgically at the time of Glenn anastomosis. Imaging (Figure 1) showed good positioning of the stent in the IVC at the right atrial junction without obstruction.

**Figure 2:**
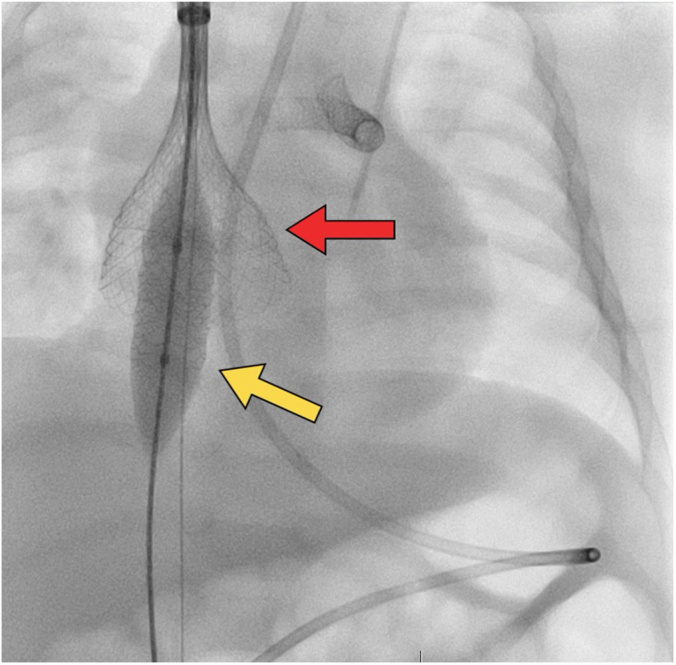
**The ŌNŌ retrieval device (red arrow) is shown deployed around the balloon holding the atrial septal defect stent in place (yellow arrow)**.

**Figure 3:**
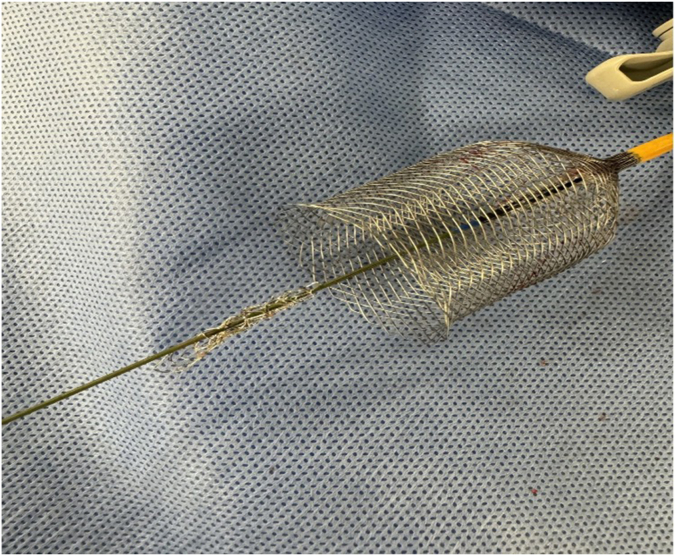
**The ŌNŌ retrieval device ex vivo with the collapsed stent on the wire**.

After further discussion with the surgical team, the decision was made to attempt retrieval of the dislodged stent with the ŌNŌ device, as we felt that the leaving the stent in situ would compromise IVC flow for a future Fontan circulation. After angiographic assessment of the right superior vena cava, access was obtained via the right internal jugular vein with a 14F Dryseal sheath (Gore). The ŌNŌ device requires a minimum 12F sheath, which we would have preferred to use a 12F sheath; however, a 14F catheter was the smallest we had in our supply at the time. Femoral venous access was also obtained and a venovenous rail was created between the femoral and jugular veins. This would have prevented stent migration in the case the ŌNŌ did not capture the stent. A 12.0-mm Mustang Balloon Dilation Catheter (Boston Scientific) was advanced from the right femoral venous sheath placed within the stent; this was used to push the stent up into the body of the right atrium, as well as hold it in place for easier capture with the ŌNŌ (the ŌNŌ has a 7F lumen, and the balloon could be advanced through the ŌNŌ). The ŌNŌ device was then advanced through the Dryseal sheath and expanded over the balloon and stent (Figure 2). The ŌNŌ was then gently retracted with simultaneous slow deflation of the balloon, collapsing the stent in the process. The ŌNŌ system was removed from the Dryseal sheath with the stent encapsulated within the device (Figure 3). The infant tolerated the retrieval procedure well and without further complications.

## Discussion

Retrieval of endovascular foreign bodies requires careful technique. Surgical retrieval and endovascular retrieval with snares are typically used. Typically, only relatively small or easily collapsable objects can be retrieved through catheters. Stents are typically treated as permanent—retrieval of fully deployed stents is challenging and poses a risk of endothelial injury or dissection.^1^ In this case report, we presented a case of the youngest known patient to date to undergo a transcatheter retrieval using the ŌNŌ device, at an age of 4 months 30 days and weight of 6.24 kg. He underwent endovascular removal of an atrial stent after it was dislodged from the atrial septum. Previous reports demonstrated safe and effective retrieval of a variety of endovascular foreign bodies in adult patients, including a MitraClip (Abbott Vascular), Micra leadless pacemaker (Medtronic), and a covered CP stent (NuDEL).^2^, ^3^, ^4^ Additionally, the ŌNŌ device has been used to remove cardiac tumors and vegetation, highlighting its potential in treating a range of cardiac conditions.^5^^,^^6^ Before this report, the youngest known patient who underwent catheterization using the ŌNŌ device was a 14-year-old boy who had a subacute intracardiac thrombus.^7^ Use of the ŌNŌ device in this case was essential to avoid surgery as the alternative means of stent removal. The use of this minimally invasive device provided a safe and effective method for stent removal and avoiding major surgery.
